# Multiorgan failure and death from a mixed Dettol and Clorox poisoning: a case report

**DOI:** 10.1186/s13256-023-03825-0

**Published:** 2023-03-16

**Authors:** David Olukolade Alao, Kinza Moin, Snaha Abraham

**Affiliations:** 1 Department of Internal Medicine, College of Medicine and Health Sciences, United Arab Emirates University, Al Ain, United Arab Emirates; 2 Department of Emergency Medicine. Tawam Hospital, Al Ain, United Arab Emirates

**Keywords:** Dettol, Sodium hypochlorite, Poisoning, Multi-organ failure, Death

## Abstract

**Background:**

Dettol and sodium hypochlorite have wide use as household disinfectants and cleaners. Intentional and nonintentional ingestion are widespread, mainly causing mild symptoms that require no specific treatment. However, severe complications can occur when large volumes are ingested. Both products affect the same organ systems in the body, which can result in fatalities when ingested together.

**Case presentation:**

We present the case of a 26-year-old Asian man who died from multiorgan failure after deliberately ingesting a presumed large volume of Dettol and sodium hypochlorite. The case illustrates the severe complications that can occur with mixed ingestion of these commonly used household products.

**Conclusion:**

Clinicians must be aware of the increased risk of death caused by the combined ingestion of chloroxylenol and sodium hypochlorite.

## Background

Dettol is a widely used household disinfectant composed of chloroxylenol, pine oil, and isopropyl alcohol. Nonintentional and intentional ingestion are widespread, causing mild symptoms that require no specific treatment. Exposure to household cleaners and disinfectants was the second highest (8.37%) reason for all the calls made to poison centers in the USA in 2021. Fatalities following ingestion were very low, with only 1434 deaths reported following over 2.5 million exposures [1]. However, major complications have been reported in up to 8% of cases [2]. Previously reported deaths from Dettol poisoning are mainly due to upper airway compromise as a result of reduced consciousness level, aspiration complications, and acute respiratory distress syndrome (ARDS) [2].

Sodium hypochlorite is the active component of Clorox, a common household cleaner. It also has use in root canal surgery in dentistry. Poisoning from sodium hypochlorite ingestion is common, with an average of over 44,000 enquiries per year made to poison centers in the USA [3]. The reported cases were mainly of nonintentional consumption, and few were associated with complications and death [4, 5]. Ingestion of Dettol and sodium hypochlorite are common in our setting, but the actual incidence is unknown because we do not have a national poison data service.

We present the case of a 26-year-old Asian man who died from a severe gastrointestinal hemorrhage and multiorgan failure following the deliberate ingestion of a large quantity of chloroxylenol and sodium hypochlorite. The case illustrates the severe complications that can occur when these commonly used household products are ingested together.

## Case presentation

The emergency medical service (EMS) brought a 26-year-old Asian man to the emergency department (ED) approximately 2 hours after ingesting Clorox (sodium hypochlorite) and Dettol from two separate 1-l bottles. His workmate had found him “shaking” in the bathroom and had called for an ambulance. He was distressed and confused, with increased oral secretion and drooling. The EMS started him on intravenous normal saline and oxygen via a nonbreather mask.

On examination in the ED, the patient complained of abdominal pain and headache. His initial vital signs were respiratory rate of 22 breaths per minute, oxygen saturation 98% on a non-rebreather mask, heart rate of 84 beats per minute, blood pressure (BP) 182/102 mmHg, and Glasgow Coma Scale (GCS) was 13/15 (E4, V4, M5). The patient’s oral mucosa and oropharynx were erythematous with slough and moderate swelling. He was drooling but had no stridor. He had drug-assisted endotracheal intubation using direct laryngoscopy and a size 7.5 mm tube, based on suspicion of an impending airway compromise. He was put on a mechanical ventilator in continuous mandatory ventilation (CMV) mode. A blood sample was collected for venous gas, full blood count (FBC), urea and electrolytes (U and E), clotting screen, liver functions, and drug toxicology screen.

Bedside and admission blood tests showed venous blood gas (VBG) pH 7.26 (7.35–7.45), CO_2_ 36.9 kP (33–45), HCO3 16 mEq/l (22–28), Base excess (BE) −10 (−2 to +2), lactate 4.5 (< 2). White blood cell count (WBC) was 16.9 (4–9) with neutrophilia. International normalized ratio (INR) was I.4 (1.0–1.1), with prothrombin time (PT) and activated partial thromboplastin clotting time (APTT) of 14.3 (11.5–15.55) and 39.7 (30–40), respectively. There was evidence of hepatic and pancreatic injury with raised aspartate aminotransferase (AST) 122 mEq/l (0–20 mEq/l) and alanine transaminase (ALT) 74 mEq/l (0–20 mEq/l). His amylase and lipase levels were 1137 U/l (25–125 U/l) and 3000 U/l (25–60 U/l). Urine and serum toxicology screens, including phosphatidyl ethanol levels, were negative.

**Fig. 1:**
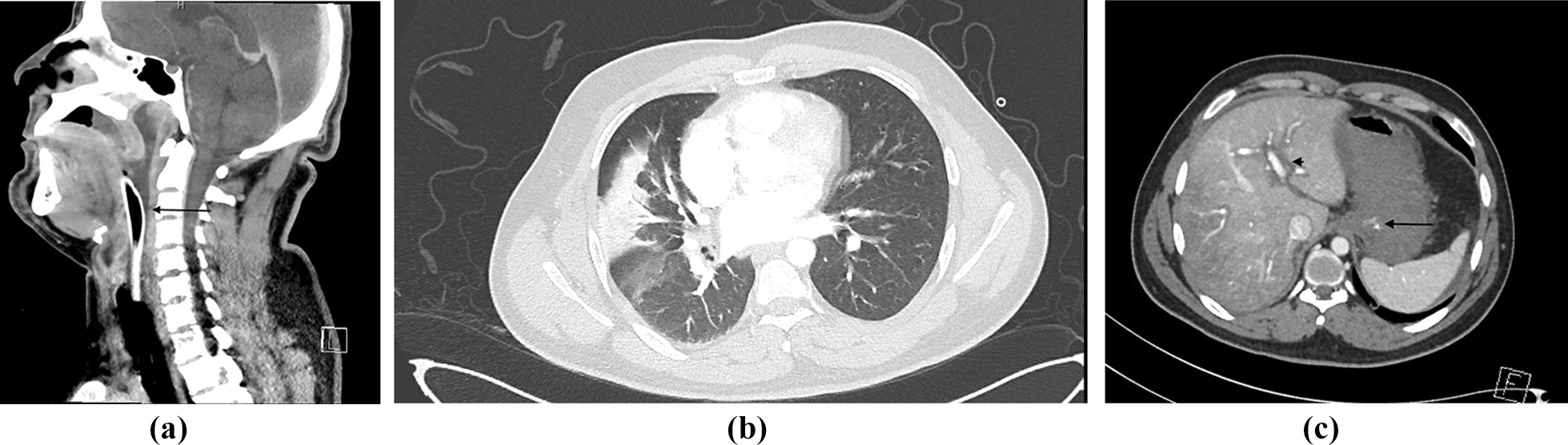
**a** Computed tomography of the neck with an arrow showing retropharyngeal swelling. **b** Computed tomography of chest showing aspiration pneumonitis in the right lung. **c** Computed tomography of the abdomen with an arrowhead showing periportal swelling and arrow showing the stomach filled with blood with associated contrast blush

A chest X-ray confirmed the correct position of the endotracheal tube and a right middle lobe consolidation, suggestive of aspiration pneumonitis. A contrast computed tomography scan of the head, neck, thorax, and abdomen showed (a) significant soft tissue edema of the larynx and pharynx, (b) ground glass opacity right lower and middle lobes, and (c) blotch of contrast in the stomach fundus, peripancreatic and perigastric streaks with contrast substance extravasation in the stomach, and hypodensity of the periphery of the liver in mainly the right lobe and periportal edema. Figure 1a–c show the computed tomography of the larynx, chest, and abdomen.

**Fig. 2:**
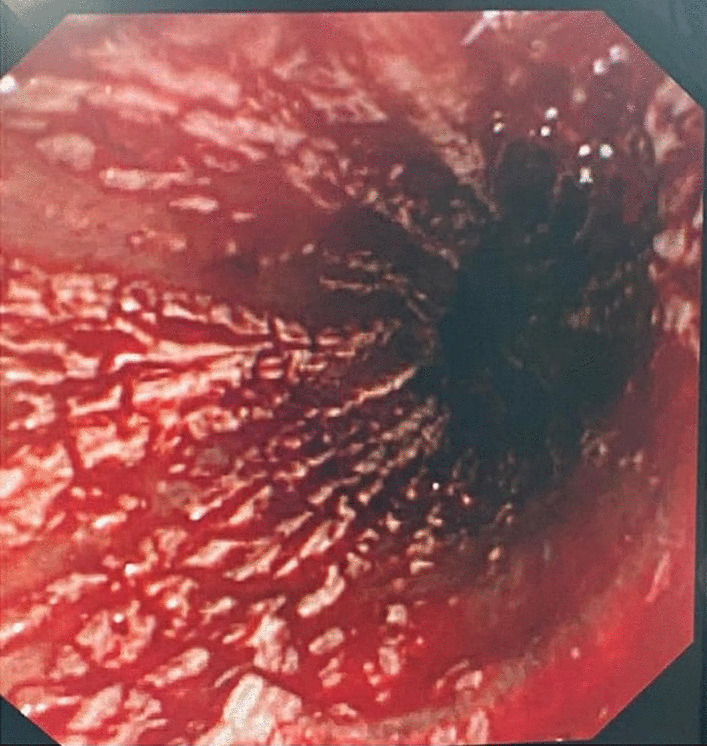
Endoscopy showing severe esophageal chemical erosion

An esophagogastroduodenoscopy (OGD) showed severe erosive esophagitis with blood oozing throughout. The gastric lumen was filled with blood, obscuring the view of the stomach wall and duodenum (Fig. 2). The patient was admitted to the intensive care unit.

On the 2nd day of admission, the patient’s heart rate was 147 beats per minute, and his BP was 76/55 mmHg. Repeated laboratory results showed worsening hepatic and renal functions and raised procalcitonin levels.

The patient was diagnosed with sodium hypochlorite and Dettol-induced corrosive gastrointestinal mucosa injury on the basis of the OGD findings.

His laboratory test results showed a high anion gap metabolic acidosis and hyperkalemia, indicating acute kidney injury. He developed multiorgan failure involving the lungs, pancreas, and liver and disseminated intravascular coagulopathy (DIC).

The patient received a loading dose of intravenous pantoprazole 80 mg, followed by a continuous infusion at a rate of 8 mg per hour. Intravenous normal saline was continued, and he was started on intravenous piperacillin/tazobactam 4 g/0.5 g. The patient was catheterized to monitor urine output, and frank hematuria was noted.

His DIC was treated with transfusion with factor VIIa recombinant, fresh frozen plasma, and cryoprecipitate.

He was placed on sodium bicarbonate infusion and continuous renal replacement therapy due to worsening metabolic acidosis and hyperkalemia. Intravenous vancomycin 1 g was added for broader antimicrobial coverage.

The patient had persistent hypotension, which was treated with norepinephrine, vasopressin, and packed red blood cells (RBC) transfusion. In total, he was transfused with 12 units of packed RBCs, 23 packs of fresh frozen plasma, three packs of cryoprecipitate, and three units of platelets.

He had persistent metabolic acidosis and hyperlactatemia, despite renal replacement therapy and continuous bicarbonate infusion.

On day 5 of post-hospital admission, the patient’s condition remained critical with worsening bradycardia (heart rates of 30–47 beats per minute) and invasive mean arterial blood pressure of 30–40 mmHg. His laboratory values on day 5 were creatinine 100 mmol/l, alkaline phosphatase 441 IU/l, AST > 7000 IU/l, ALT 2820 IU/l, N-terminal (NT)-pro hormone brain natriuretic peptide 32 (NT pro-BNP) 477 ng/l, troponin > 10,000, hemoglobin (Hb) 83 g/l, platelets 24 × 10^9^/l, pH 7.19, base excess −17.30, HCO_3_ 12, and lactate 18 mmol/l. The patient was placed on a do not attempt resuscitation (DNAR) order in case of cardiac arrest following a review by the ICU multidisciplinary team comprising intensivists, renal physicians, gastroenterologists, and representatives of the patient’s family. The patient subsequently went into cardiac arrest and was pronounced dead.

## Discussion

The patient died of multiorgan failure. This case highlights the additive effects of the combined ingestion of sodium hypochlorite and Dettol on body systems, especially the gastrointestinal, renal, cardiac, and hepatic systems. Chan, T.Y. and his colleagues reported an incidence of only 5.6% gastrointestinal bleeding in their study of 89 cases of Dettol poisoning. All were mild and required no management [6]. This is the first reported case of multiorgan failure and death from combined Dettol and sodium hypochlorite ingestion.

Dettol is a mixture of chloroxylenol (4.8%), pine oil (9%), and isopropyl alcohol (12%). Poisoning from Dettol is widespread. Patients who ingest less than 60 ml are asymptomatic. The most typical symptoms following ingestion are vomiting (57%), throat pain (38%), and dizziness (27%) [7]. These symptoms reflect the local and systemic effects of the constituent. Ingestion of 200 ml or more can result in renal impairment and death [7, 8].

Clorox contains 3–6% sodium hypochlorite (NaOCl) and sodium carbonate (0.20%). When sodium hypochlorite is ingested, it produces hypochlorous acid (HOCl), which rapidly converts to hydrochloric acid (HCl) and oxygen radical (O^−^). Both chemicals can cause cellular protein damage and cell death. The effects of these chemicals in the body are related to the quantity ingested. Patients who ingest less than 120 ml of 3–6% concentration will experience little or no symptoms [9]. Corrosive gastroesophagitis and systemic symptoms are expected in adult patients who ingest 150–200 ml of 3–6% sodium hypochlorite solution [4, 5]. Death has been reported following ingestion of 750 ml, and intentional ingestions should be suspected in such cases [10]. Table 1 illustrates the additive effects of these two products on the body systems.

**Table 1:** Clinical symptoms caused by Clorox and Dettol, by body system

Chemical agents	Clinical symptoms					
	GIT	CVS	CNS	Respiratory	Renal	Metabolic
Clorox						
Sodium hypochlorite	Vomiting diarrhea corrosive burn			Pneumonitis ARDS		Acidosis electrolytes derangement
Dettol						
Chloroxylenol		Arrhythmia	Depression seizure	Depression	Nephrotoxic	
Pine oil	Irritation corrosive burn	Hypotension	Depression	Pneumonitis ARDS		
Isopropyl alcohol	Vomiting hematemesis	Hypotension	Depression	ARDS	Nephrotoxic	

**Fig. 3:**
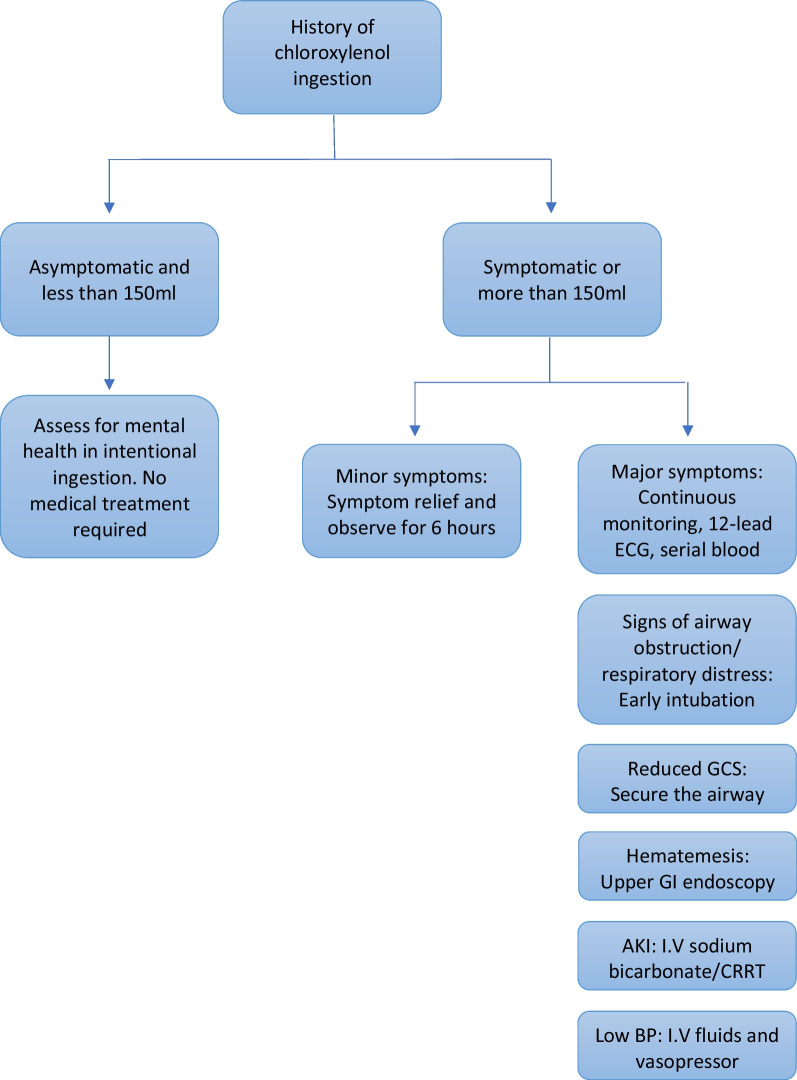
Flow chart for the management of chloroxylenol poisoning (drawn by the first author)

Management of Dettol poisoning depends on various factors, such as the route of ingestion, the time elapsed since the ingestion, and the volume ingested. Figure 3 provides a flow chart for the management of Dettol poisoning.

Patients with severe poisoning need continuous monitoring of vital signs, electrocardiogram (ECG), and serial blood gas analysis for at least 24 hours [4].

Maintenance and protection of the airway and adequate ventilation is the first step in managing Dettol poisoning in patients with reduced consciousness levels [4]. Early intubation should also be done if there are signs of upper respiratory tract obstruction, respiratory distress, stridor, erythema, and burn in the supraglottic area.

Renal impairment should be managed with intravenous sodium bicarbonate and early hemodialysis. A multidisciplinary approach from gastroenterologists and otolaryngologists is essential after the initial stabilization of the patient.

Gastric lavage is harmful due to the increased risk of aspiration. Upper gastrointestinal endoscopy has prognostic value and should be done within 24 hours to determine the extent of the corrosive injury [11]. However, endoscopy is contraindicated in hemodynamically unstable patients and those with signs of perforation or airway compromise. Death can occur in up to 2% of Dettol poisoning cases, and is associated with upper respiratory tract obstruction [4].

**Fig. 4:**
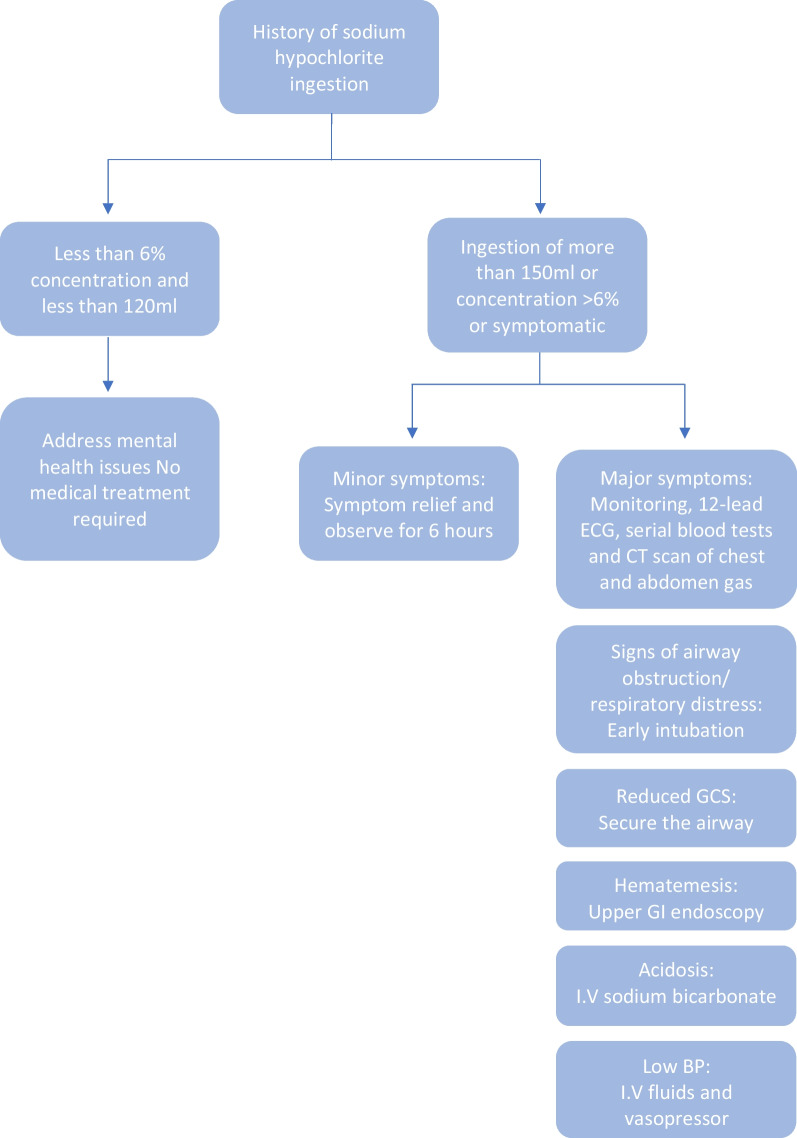
Flow chart for the management of sodium hypochlorite poisoning (drawn by the first author)

Figure 4 provides a flow chart for the management of sodium hypochlorite poisoning. Flexible endoscopy and computed tomography of the chest and abdomen are essential for assessing severity, mortality, and stricture formation risk [10]. Mortality from sodium hypochlorite poisoning is rare, with only one reported death in nearly 22,000 exposures over 3 years [11–13].

Multiorgan failure resulting from the ingestion of household cleaning products is uncommon. To the best of our knowledge, we are unaware of any previous report of multiorgan failure of the liver, pancreas, and kidney leading to death due to combined ingestion of these common household products.

## Conclusions

Poisoning from sodium hypochlorite and Dettol is common, and severe morbidity such as airway compromise, aspiration pneumonitis, corrosive gastrointestinal injury, and depressed consciousness level can occur when greater than 150 ml of either chemical is ingested.

Previous studies have reported death due to airway compromise, while gastrointestinal bleeding tends to be mild. This report shows that combined ingestion of a large volume of Dettol and sodium hypochlorite can cause severe gastrointestinal bleeding, multiorgan failure, and death.

## Data Availability

None.
